# Whooping Cough

**Published:** 1879-08

**Authors:** 


### WHOOPING COUGH.

A novel method of treating these cases is given in the St. Louis *Medical Journal.* He charges a steam atomizer with the following mixture:

| | | |
|---|---|---|
| ℞ | Ext. Belladonnæ Fluid, - - - | gtt vi to xii |
| | Ammonii Bromidi, - - - - - | ℈i |
| | Potass Bromidi, - - - - - - | ℈ii |
| | Aq: Destillat, - - - - - - | ℥ii |

Misce.

The atomizer being placed on a table before the patient, the spray is rapidly carried over into the face, mouth and lungs of the child, and continued 10 or 15 minutes till the pupils are dilated by the effect of the Belladonna mixture. The application is to be made morning, noon and at bed-time. This has so far cut short the spasmodic cough within two or three days, uniformly and almost to a certainty.

